# CircDiaph3 influences PASMC apoptosis by regulating PI3K/AKT/mTOR pathway through IGF1R

**DOI:** 10.1007/s13205-023-03739-0

**Published:** 2023-09-11

**Authors:** Ge Liu, Shengqiang Zhang, Shaofeng Yang, Chongwen Shen, Chao Shi, Wenjie Diao

**Affiliations:** Department of Cardiac Surgery, The First Affiliated Hospital of Bengbu Medical College, Bengbu, Anhui People’s Republic of China

**Keywords:** Pulmonary arterial hypertension, circDiaph3, Smooth muscle cells, Hypoxia, Insulin growth factor signaling

## Abstract

The pathogenesis of pulmonary hypertension has not been elucidated. We investigated the role of a circular ribonucleic acid, circDiaph3, in the proliferation and migration of pulmonary artery smooth muscle cells during pulmonary hypertension. CircDiaph3 overexpression in blood samples of patients with pulmonary hypertension was analyzed by real-time quantitative polymerase chain reaction. Subsequently, a rat model of pulmonary arterial hypertension was established under hypoxic conditions. Pulmonary artery smooth muscle cells were harvested from the rat model for subsequent experiments with small interfering ribonucleic acid-mediated knockdown of circDiaph3. In cell model, we found that PI3K, AKT, mTOR and insulin-like growth factor 1 signaling pathway (IGF1R) and smooth muscle cell marker genes (α-SMA, Vcam1) were significantly downregulated. The overexpression of *Igf1r* in pulmonary artery smooth muscle cells rescued the downregulated smooth muscle cell genes, IGF1R signaling pathway proteins, increased smooth muscle cell proliferation, and reduced apoptosis. CircDiaph3 regulates the PI3K/AKT/mTOR signaling pathway via IGF1R to inhibit apoptosis and promote proliferation of smooth muscle cells. Additionally, adenovirus-mediated in vivo inhibition of circDiaph3 was carried out in rats with pulmonary arterial hypertension, followed by harvesting of their pulmonary artery smooth muscle cells for subsequent experiments. Excessive proliferation of smooth muscle cells in the pulmonary artery has narrowed the pulmonary artery lumen, thereby causing pulmonary hypertension, and our results suggest that circDiaph3 has important value in the treatment of pulmonary hypertension.

**Supplementary Information:**

The online version contains supplementary material available at 10.1007/s13205-023-03739-0.

## Background

Pulmonary arterial hypertension (PAH) is a chronic disease affecting the heart and lungs. PAH is caused by excessive proliferation and fibrosis in the wall of the pulmonary artery. Due to fibrosis, the vascular lumen of the pulmonary artery is reduced leading to an increased pulmonary vascular resistance (PVR) (Humbert et al. 2014). Consequently, pressure on the heart increases leading to heart failure. The global incidence of PAH is in several millions (Rich, et al. 2018). Some of the disease conditions leading to PAH include chronic obstructive pulmonary disease, thromboembolism, and heart disease (Hoeper et al. 2016).

The pathogenesis of PAH differs according to its etiology (Tuder et al. 2009). However, some of the common pathological events during PAH include excessive constriction and remodeling of the pulmonary artery, which leads to a narrowed lumen of the pulmonary artery, decreased pulmonary compliance, and increased right ventricular afterload (Hassoun 2021). Although pathological events, such as endothelial cell injury, excessive proliferation of endothelial cells, and invasion of fibroblasts, all occur in the PAH pathogenesis, the proliferation of pulmonary artery smooth muscle cells (PASMCs) is the most significant feature of PAH (Galiè et al. 2019). The pulmonary artery remodeling during PAH involves a chronic inflammatory response, which leads to an increase in the levels of interleukin (IL)-1, IL-8, IL-6, and matrix metallopeptidase 9 (Ou et al. 2020).

Circular ribonucleic acids (circRNAs) are a type of non-coding RNA. circRNAs are produced by backsplicing of protein-coding gene exons, sometimes with introns, in eukaryotic cells (Li et al. 2018). circRNAs are mainly present in the nucleus and cytoplasm, and their sequences are highly conserved and more stable than the linear RNAs (Gao et al. 2018). In addition, previous reports suggest that a variety of circRNAs are involved in the proliferation and migration of PASMCs, which could indicate their role in the development of PAH (Su et al. 2020).

In this study, we investigated the role of a circRNA, circDiaph3, in the proliferation and migration of pulmonary artery smooth muscle cells during pulmonary hypertension. We detected circDiaph3 in the blood of PAH patients. Our data from the primary culture of PASMCs of rats with PAH showed that circDiaph3 plays an important role in promoting the proliferation of PASMCs through insulin-like growth factor 1 receptor (IGF1R) and phosphatidylinositol-3-kinase/protein kinase B/mammalian target of Rapamycin (PI3K/AKT/mTOR) signaling pathway. In addition, our data also indicated that circDiaph3 regulates the expression of PASMC marker genes (*α-SMA, Vcam1*) required for proper development and functioning of PASMCs.

## Methods

### Patient description and sample collection

This study was approved by the Ethics Committee on Human Experiments of Bengbu Medical College (Protocol number 2022279), and all participants signed a consent form before inclusion in the study. Fresh blood samples were collected from 15 patients, who were diagnosed with PAH (pulmonary arterial pressure > 25 mmHg) between January 2022 and June 2022 at the Department of Cardiac Surgery of the First Affiliated Hospital of Bengbu Medical College of China. PAH was diagnosed according to echocardiographic, which was defined as a mean pulmonary arterial pressure ≥ 25 mmHg. Exclusion criteria were (a) patients who were younger than 18; (b) patients whose PAH was reversed after CHD correction; and (c) patients with incomplete clinical data. The procedure was conducted as per the guidelines outlined in the Declaration of Helsinki. Written informed consent was obtained from each patient participating in the study. The experimental protocol and informed consent were approved by the Ethics Committee of Bengbu Medical College (Bengbu, Anhui, China). All blood samples were stored at − 80 °C for subsequent experiments.

### Establishment of animal models of PAH and adenovirus injection

#### Hypoxia-induced PAH model

A total of 32 healthy Sprague Dawley (SD) rats (specific pathogen-free grade), weighing 160–200 g were obtained from the Experimental Animal Center of Bengbu Medical College.8 rats were placed in a normoxic environment as sham group, and the other 24 rats were placed under hypoxic conditions (10% O_2_) for 30 days to induce PAH in rats, Twenty-four rats were divided into three groups with eight rats in each group (control, sh-NC, sh-circDiaph3 group). The heart and lung tissue samples were extracted from control rats and those with PAH under anesthesia (Isoflurane 50 mg/kg). Subsequently, these tissue samples were placed in cold phosphate-buffered saline (PBS) until further experiments.

All procedures, experiments, and housing of the animals were carried out according to current regulations and guidelines for animal welfare and to international principles of laboratory animal care, following the ARRIVE Guidelines Checklist as well. Ethics committee of Bengbu Medical College approved this animal study. The protocols were approved by the Ethics Review Board of Bengbu Medical College, Bengbu, Anhui Province, China (Protocol number 2022356).

### PASMC primary cell culture and hypoxia treatment

PASMCs were isolated from the pulmonary arteries of rats with PAH and control rats. The isolated PASMCs were cultured with 10% fetal bovine serum at 37 °C in moist air containing 5% CO_2_ for all experiments. Immunofluorescence staining for α-smooth muscle actin (α-SMA) was used to ascertain the purity of cultured PASMCs.

The PASMCs of control rats with PAH were cultured in humid air containing 5% CO_2_ at 37 °C for 24 h. These cells were then cultured under hypoxic conditions in a hypoxia incubator (YCP-80/S, CHAOHONG, China), with 3% O_2_/5% CO_2_/92% N_2_.

### Small interfering RNA (siRNA) transfection

The siRNA duplexes for gene silencing were obtained from HANBIO. The siRNA sequences are as follows: (1) circDiaph3 (si-circDiaph3) — 5′-GUUUGUAACCAUGUGUGUGUA-3′ and 5′-UACAGAGACAUGGUUACAAAC-3′; 2) scrambled siRNA as negative controls (si-Con) — 5′-UUCUCCGAACGUGUCACGUTT-3′ and 5′-ACGUGACACGUUCGGAGAATT-3′.

### Quantitative real-time polymerase chain reaction (qRT-PCR) analysis of gene expression

The tissue and blood were weighed and dissolved in 1 ml TRIzol (15,596,018, Thermo Fisher Scientific, China). After centrifugation at 4 °C and 12,000 rpm for 10 min, the supernatant was collected and mixed with 0.5 mL isopropyl alcohol (2,019,121,060, Zhiyuan Chemical Reagent Co., LTD, China). The RNA pellets were centrifuged at 12,000 rpm at 4 °C for 5 min. The supernatant was discarded, and the RNA pellets were air-dried at 20 °C. The following primers were used in this study: IGF1R forward, 5′-GTCAAGGACGAGCCTGAAAC-3′ and IGF1R reverse, 5′-TGAGGCCTCGTTGAGAAACT-3′; CircDiaph3 forward, 5′-GCGACAGAAGAAAAAGGACACC-3′ and CircDiaph3 reverse, 5′-GTAGAAGCTGCCTTCCCATCT-3′; PI3K forward, 5′-CGAAACAAAGCCGAGAACCT-3′ and PI3K reverse, 5′-GACGCAATGTTTGACTTCGC-3′; AKT forward, 5′-CAGGTTCACCCAGTGACAAC-3′ and AKT reverse, 5′-CTCCTTCACCAGGATCACCT-3′; and mTOR forward, 5′-AGAACCACATGCCACACAGT-3′ and mTOR reverse, 5′-CTTTGGCATTTGTGTCCATC-3′.

### Western blot analysis

The tissues and cells were weighed and incubated with 1 mL of RIPA cell lysate buffer (BL504a, Bio Sharp, USA). After centrifugation, the supernatant was collected, and sodium dodecyl sulfate (S8010, SolarBio, China) was added to the microspheres. Next, samples (30g) were loaded onto an acrylamide gel (5%). The samples were run in the stacking gel at 80 V for approximately 30 min. When the samples reached separating gel, the voltage was set at 120 V for 1 h to facilitate the protein separation process. Before the transfer, the filter paper and polyvinylidene fluoride (PVDF) membrane (IPVH00010, Millipore, USA) of the same size as the acrylamide gel were immersed in the transfer buffer for 5 min. The separated proteins were then transferred to the PVDF membrane at a constant current of 300 mA. The PVDF membrane with proteins was incubated overnight with diluted primary antibodies with slow shaking at 4 °C. The primary antibodies were washed next morning, and the PVDF membrane was incubated with horseradish peroxidase (HRP)-labeled secondary antibodies at room temperature for 2 h. The film was placed in an automatic exposure meter in a dark room, and the image was obtained under appropriate exposure conditions.

### Cell proliferation assay using cell counting Kit 8 (CCK8)

PASMCs isolated from rats with PAH and control rats were cultured in fetal bovine serum medium (CM-0122, ProCell, China). Subsequently, PASMCs were digested with trypsin (C0201, Beyotime, China) upon reaching the logarithmic growth phase, and collected with centrifugation. The cell pellet was then resuspended in the medium (with gentle mixing) to obtain a cell suspension with 5–10 × 10^4^ cells/mL. Subsequently, 100 µL of cell suspension was added to each well of a 96-well flat-bottom plate. Wells on the edge of the plate were filled with sterile PBS. The cells were incubated in the plate (5%CO2, 37 °C) until a monolayer of cells covered the bottom of the well. These cells were then incubated overnight in a low-oxygen incubator. These cells were transfected with siRNA next morning, after observing their morphology under an inverted microscope. After 48 h of culture, 10-µL CCK8 (BB-4202–01, Bebo, China) was added to each well. After incubation for 1 h, the absorbance of each well was measured at 450 nm. Data were standardized with wells containing only the medium and CCK8 as controls.

### Cell apoptosis assay

PASMCs adhered to the wall and were digested with trypsin without EDTA. After cell counting, 0.5 × 10^6^ cells were collected and centrifuged at 1500 rpm for 3 min. The cell pellet was then washed twice with PBS by centrifuging at 1500 rpm for 3 min. After washing, the cells were resuspended in 100 µl 1 × Binding buffer. In this cell resuspension, 5-µL fluorescein isothiocyanate was added, and cells were incubated in the dark for 15 min. Afterward, 10-µL propidium iodide was added to the cell suspension, and the cells were incubated at room temperature for 5 min in the dark and then put on the FCM (CytoFLEX, BECKMAN, USA) for detection.

### Cell cycle assay

The PASMCs were washed with cold PBS, followed by digestion with trypsin. The digested cells were centrifuged at 2000 rpm for 5 min and washed with cold PBS. Afterward, the supernatant was carefully removed, leaving behind a cell suspension of about 50 µL. Next, cells were resuspended into 0.3 mL PBS to obtain a single-cell suspension and 2.7 mL cold anhydrous ethanol was added to the cell suspension at a final concentration of 90%, and the cells were incubated at 4 °C overnight (18–24 h) to facilitate cell fixation. This was followed by washing the cells with cold PBS twice at 2000 rpm for 5 min. The cells were then resuspended in 500-µL PBS in a 1.5-mL Eppendorf tube and mixed gently to avoid cell clumping. Next, the cells were incubated with 20 μL RNase (storage concentration 25 mg/mL, PBS diluted to 1 mg/mL, 50 µg/mL), in a water bath at 37 °C for 30 min. The cells were then incubated with 400 µL of propidium iodide at 4 °C in darkness for 30–60 min and tested on the machine.

### Hematoxylin–eosin staining (HE)

After dehydration, the specimens were embedded and sectioned (thickness 6 μm), soaked in xylene (10,023,418, China) for 3 times, 15 min each time, and then dehydrated in gradient 100% and 80% ethanol (10,009,218, China) for 5 min. The sections were rinsed once with pure water and then washed with alcohol to remove the section impurities. The cells were incubated with hematoxylin (BA-4041, BASO, China) for 5 min and washed three times with pure water. Samples were incubated with 1% hydrochloric acid ethanol solution for 3–5 s and washed with pure water. Aqueous lithium carbonate was added, and the mixture was incubated for 30 s before washing with pure water. After dehydration with 80% ethanol, the cells were stained with eosin for 30 s (BA-4022, BASO, China) and then soaked in xylene for 2 min.

### Immunofluorescence assay

Tissues were fixed in 4% paraformaldehyde for 20 min and then soaked in 0.3% TritonX-100 (T9284, Sigma, USA) for 20 min at 20 °C. Tritonx-100 was removed, and tissues were washed with PBS. Goat serum (ZLI-9022, ZSBIO, USA) was incubated for 30 min, and the primary antibody was incubated for 60 min at 37 °C. The primary antibodies used were as follows: α-SMA (AF1032, Affinity, USA) and PCNA (AF0239, Affinity, USA). Afterward, the primary antibody was removed and the tissues were incubated with secondary antibody (A0516, Chinese Beyotime) at 37 °C in the dark for 30 min. Subsequently, the secondary antibody was removed, and the stained tissue was put into a fluorescence quencher and observed under a fluorescence microscope.

## Statistical analysis

The continuous variables of the normal distribution were expressed as mean ± standard deviation, and the classification variables were expressed in terms of frequency or percentage. Chi-square tests or Fisher's exact tests were used to assess categorical variables, and multivariate analyses were performed using Cox proportional risk models. Subsequently, to determine whether there was a linear association between PAH and circDiaph3, linear regression analysis was performed to fit the clinical results. All analyses were performed using R (The Re Foundation for Statistical Computing, Vienna, Austria http://www.R-project.org), and Empower Stats (http://www.empowerstats.com, X&Y Solutions, Inc., Boston, MA, USA) statistical packages are carried out. Statistical significance was set at *P* < 0.05.

Data were expressed as mean ± standard deviation and processed using SPSS 21.0 software (Chicago, IL, USA), and one-way ANOVA was used for comparison among multiple groups. Fisher test was used for comparison between the two groups. Statistical significance was set at *P* < 0.05.

## Results

### circDiaph3 expression was found to be significantly increased in the serum of PAH patients

To determine the expression of circDiaph3, blood samples were collected from patients with PAH. qRT-PCR results showed that serum circDiaph3 expression was significantly upregulated in patients with PAH in comparison with that in normotensive patients (Supplementary Table 1). In addition, there was a linear relationship between pulmonary arterial pressure and circDiaph3 expression in patients with PAH (Supplementary Fig. 1). This indicates that circDiaph3 expression is closely related to PAH.

### Establishment of a rat model of PAH and acquisition of primary cells

**Fig. 1:**
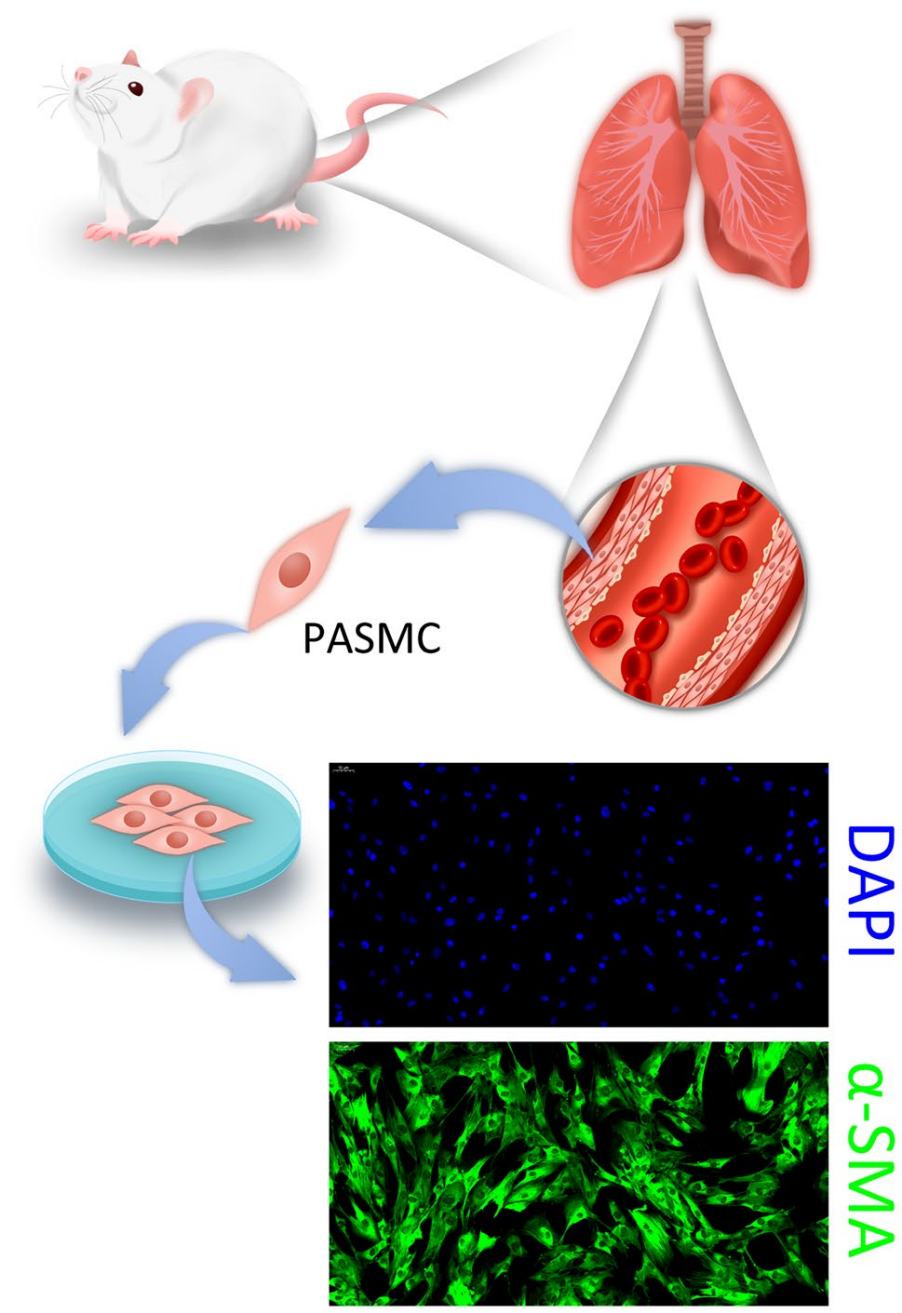
PASMC primary cells were isolated from rat pulmonary arteries after hypoxia-induced pulmonary hypertension. Pulmonary artery smooth muscle cells were labeled with α-SMA immunofluorescence staining to demonstrate successful isolation of pulmonary artery smooth muscle cells

Rats were kept in a hypoxia incubator for 30 days, which resulted in the thickening and narrowing of the pulmonary artery. Subsequently, the thickened pulmonary arteries were harvested from the rats. The vascular endothelium of the pulmonary artery was removed, and PASMCs were isolated for primary culture (Fig. 1). To assess the cell purity for PASMCs, the cultured cells were immunostained for smooth muscle marker α-SMA, which showed that the cells were rich in PASMC.

### circDiaph3 knockdown inhibited the proliferation and promoted cell death in PASMCs

**Fig. 2:**
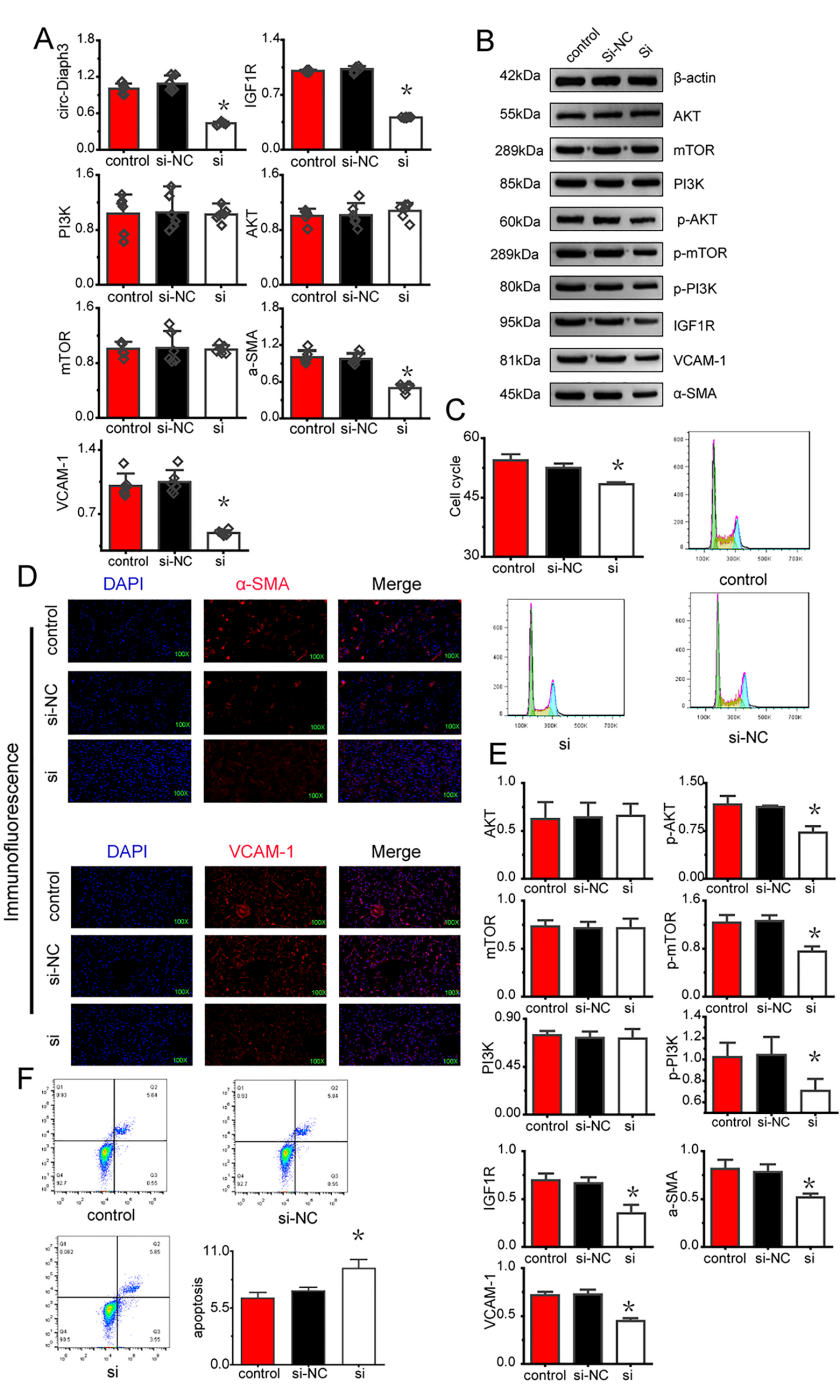
circDiaph3 inhibits the proliferation of pulmonary artery smooth muscle cells (PASMCs). The expression levels of circDiaph3, Igf1r and PI3/Akt/mTOR pathway proteins in the cells were detected by qRT-PCR and Western blot. After that, the translation of circDiaph3 was manipulated by siRNA and the expression of Igf1r and PI3/Akt/mTOR pathway was observed **A** qRT-PCR analysis of circDiaph3, *α-SMA*, *Vcam1*, *Igf1r, PI3K, Akt,* and *mTOR* in PASMCs. **C** After circDiaph3 knockdown, PASMC cycle analysis was performed by flow cytometry. **D** Immunostaining of α-SMA and VCAM1 in PASMCs post-circDiaph3 knockdown. **B**, **E** Western blot analysis of α-SMA, VCAM1, and signaling proteins of IGF-1 pathway in PASMCs. **F** Flow cytometry-based detection of PASMC apoptosis of post-circDiaph3 knockdown. Data are presented as the mean ± standard deviation; *: compared with the control group, *p* < 0.05

SiRNA-mediated knockdown of circDiaph3 in the PASMC culture resulted in decreased cell proliferation and increased apoptosis of PASMCs (Fig. 2C, F) and decreased expression of smooth muscle cell-related proteins (α-SMA, Vcam1) detected by immunofluorescence staining (Fig. 2D), in comparison with knockdown with control siRNAs. Subsequently, we checked the expression of *α-SMA*, *Vcam1*, *Igf1r*, PI3K, Akt, and *mTOR* by qRT-PCR and found that the expression levels of*α-SMA*, *Vcam1*, and *Igf1r* were significantly decreased in PASMCs post-siRNA-mediated knockdown circDiaph3 (Fig. 2A). Western blot results also showed decreased phosphorylation of AKT and mTOR and decreased protein levels of IGF1R, α-SMA, and VCAM1 (Fig. 2B, E).

### circDiaph3 interacts with PI3K, AKT, and mTOR in the cytoplasm through IGF1R

**Fig. 3:**
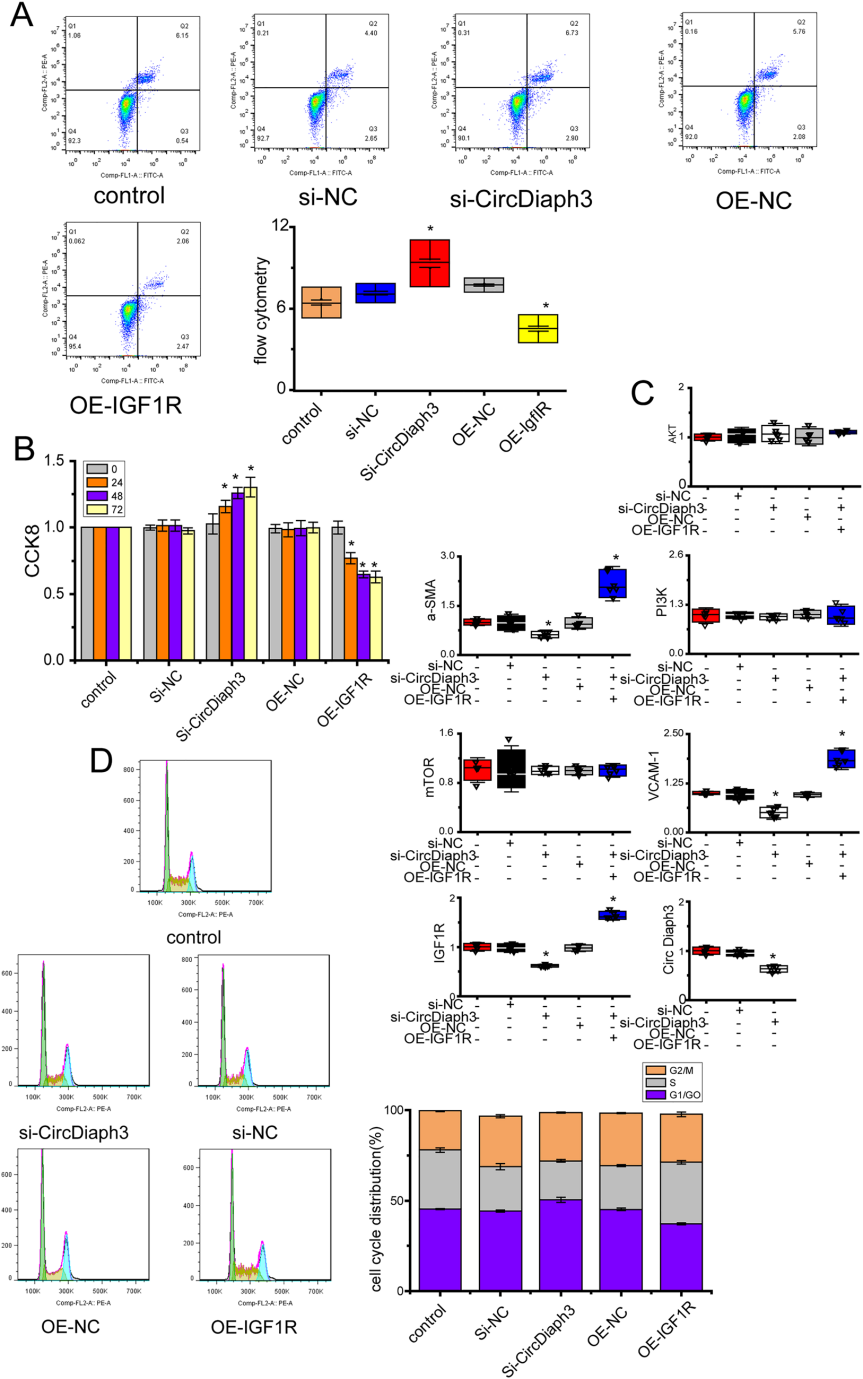
The cell cycle and the expression of related proteins were detected by flow cytometry, CCK8, qRT-PCR and Western blot. It further indicates that circDiaph3 inhibits pulmonary artery smooth muscle cell (PASMC) proliferation via *ˆIgf1r* and PI3K/AKT/mTOR signaling pathway. **A** Flow cytometry-based detection of PASMC apoptosis. **B** CCK8 assay for detecting PASMC proliferation. **C** qRT-PCR analysis of circDiaph3, α-SMA, *Vcam1*, *Igf1r, PI3K, Akt,* and *mTOR* in PASMCs. **D** PASMC cycle analysis performed by flow cytometry. Data are presented as the mean ± standard deviation (SD); *compared with the control group, *p* < 0.05

**Fig. 4:**
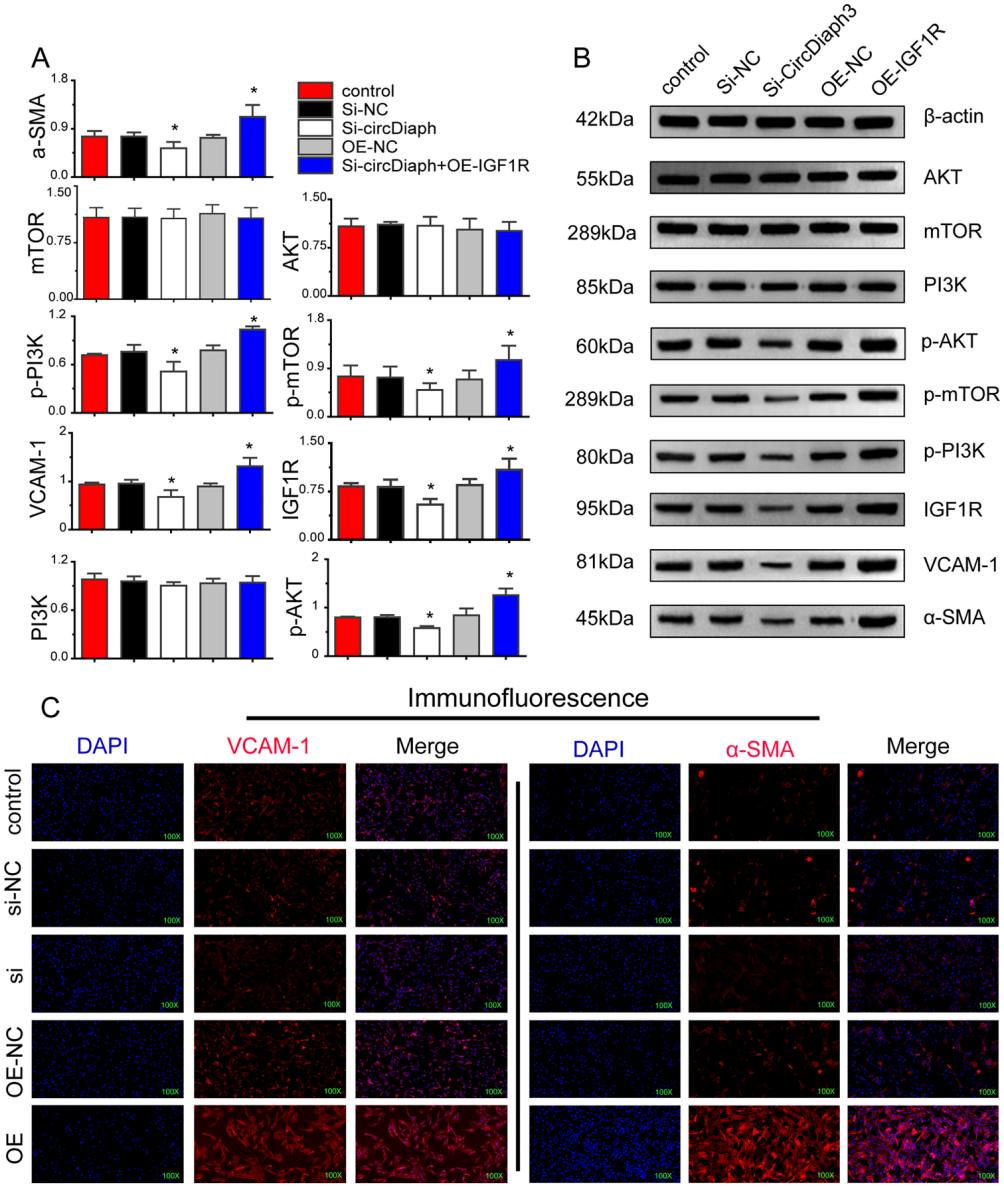
A rescue experiment was designed in the study to further prove that *Igf1r* overexpression rescues the effects of circDiaph3 knockdown in pulmonary artery smooth muscle cells (PASMCs) **A**, **B** Western blot analysis of *α-SMA*, *Vcam1*, and *Igf1r* signaling pathway proteins in PASMCs. **C** Immunostaining of *α-SMA* and VCAM1 in PASMCs. Data are presented as the mean ± standard deviation; *compared with the control group, *p* < 0.05

CircDiaph3 knockdown resulted in reduced expression of α-SMA and VCAM1, and reduced phosphorylation of PI3K, AKT, and mTOR signaling mediators of the insulin-like growth factor 1 (IGF-1) pathway (Fig. 4A and B). To check whether *Igf1r* overexpression can rescue the effects of siRNA-mediated knockdown of circDiaph3, we transfected an overexpression plasmid of *Igf1r* in the PASMCs after circDiaph3 knockdown. Indeed, post-overexpression of *Igf1r*, the PASMCs showed increased cell proliferation, reduced cell death, and change in cycle stages (Fig. 3A, B and D). In addition, the expression of smooth muscle cell markers (α-SMA and VCAM1) was found to be rescued post-*Igf1r* overexpression (Fig. 3C, Fig. 4) in PASMCs. The overexpression of *Igf1r* also rescued the levels of phosphorylated PI3K, AKT, and mTOR (Fig. 4A, B) in the PASMCs.

### circDiaph3 plays an important role in rat models of pulmonary hypertension

**Fig. 5:**
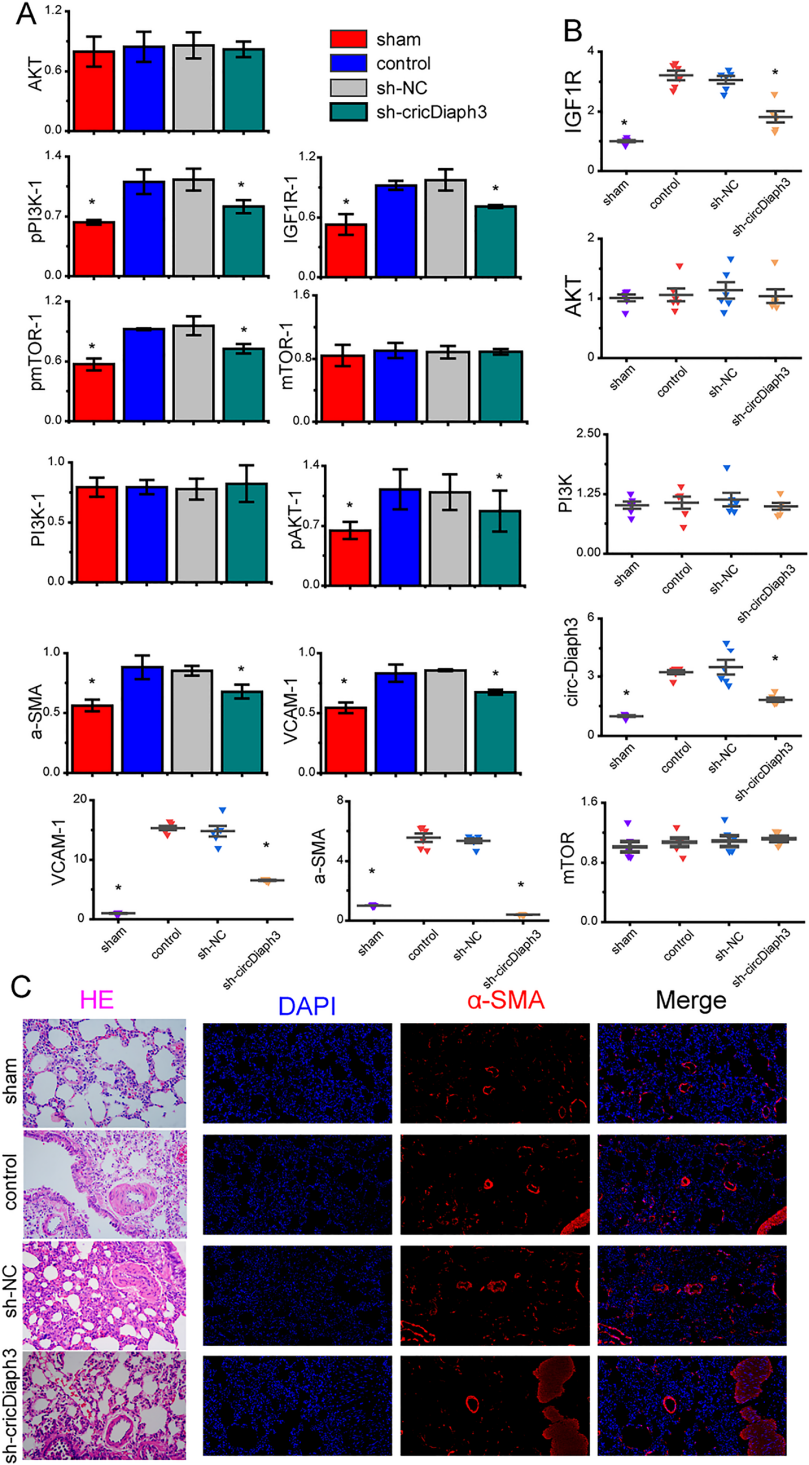
A rat model of pulmonary hypertension was established and a virus was designed to inhibit the expression of circDiaph3. Adenovirus-mediated in vivo inhibition of circDiaph3 expression in pulmonary artery smooth muscle cells (PASMCs) isolated from rats with PAH **A** Western blot analysis of *α-SMA*, *Vcam1*, and *Igf1r* signaling pathway proteins in rat pulmonary artery specimens. **B** qRT-PCR analysis of circDiaph3, *α-SMA*, *Vcam1*, and genes encoding *Igf1r* signaling pathway proteins in rat pulmonary artery specimens. **C** Hematoxylin and eosin (HE) analysis and immunofluorescence staining of *α-SMA* in rat pulmonary artery specimens. Data are presented as the mean ± standard deviation; *compared with the control group, *p* < 0.05

To understand the role of circDiaph3 in vivo, we injected an adenovirus in the PAH rats to inhibit circDiaph3 expression. We divided the PAH rats into the following groups: uninjected controls, empty virus-injected group, and adenovirus-injected group. The adenovirus-injected animals showed reduced expression of circDiaph3 in the pulmonary artery, which indicated that the virus injection and circDiaph3 inhibition were working. The adenovirus-injected animals showed reduced expression levels of α-SMA and VCAM1 and reduced phosphorylation of PI3K, AKT, and mTOR (Fig. 5A, B); Supplement Fig. 2) in the isolated PASMCs. Additionally, hematoxylin and eosin (HE) and immunofluorescence staining showed that the proliferation of PASMCs decreased significantly after circDiaph3 inhibition (Fig. 5C).

## Discussion

**Fig. 6:**
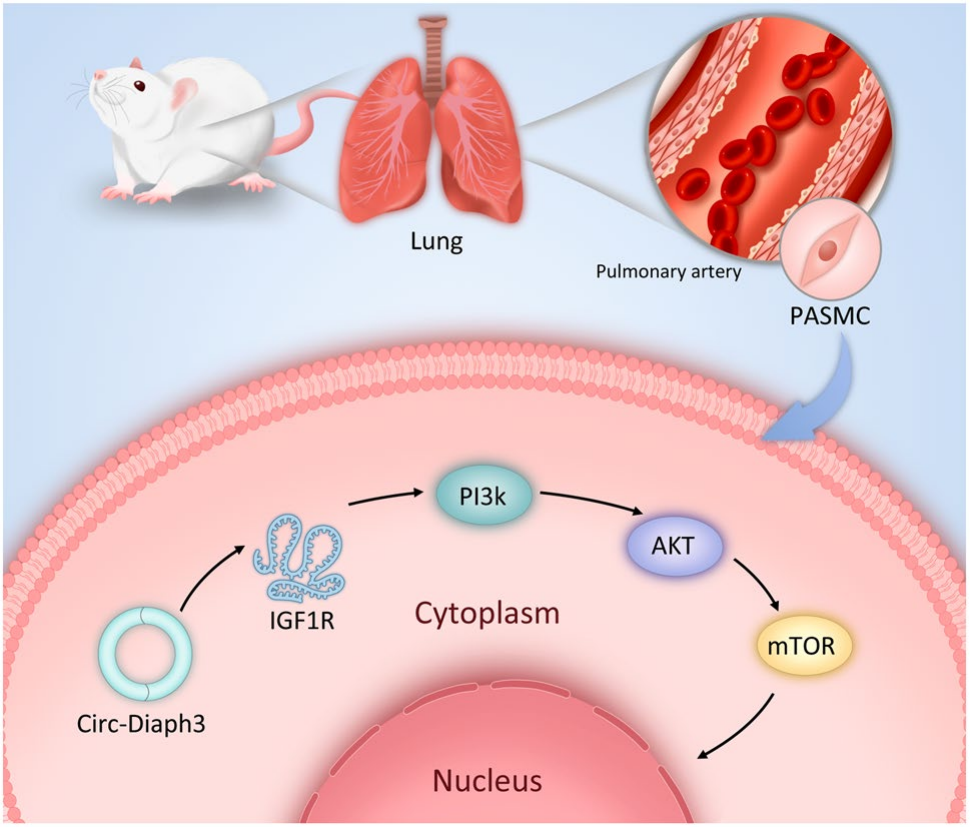
circDiaph3 promotes pulmonary artery smooth muscle cell (PASMC) proliferation through the *Igf1r* and *PI3K*/*Akt*/ *mTOR* pathway, thereby causing pulmonary hypertension

Previous studies have shown that PAH pathogenesis is closely related to the proliferation, apoptosis, and migration of PASMCs (Zhang et al. 2020). circRNAs have been shown previously to regulate genes and signaling pathways related to cell proliferation, apoptosis, and migration in PASMCs (Jiang et al. 2021). In this study, we discovered that the circDiaph3 expression was significantly upregulated in PAH patients. To confirm the role of circDiaph3 in PAH pathogenesis, we performed our studies on the following two models: (1) in vitro circDiaph3 knockdown model and (2) in vivo circDiaph3 inhibition model. Our data indicated that circDiaph3 controls PASMC proliferation and apoptosis via regulation of IGF-1 signaling (Fig. 6).

IGF-1, a member of the insulin/IGF family, plays a key role in the overall growth and metabolism of the organism and in cellular processes, such as proliferation, migration, differentiation, adhesion, and apoptosis (Macvanin et al. 2023; Steffensen, et al. 2019). IGF-1 is secreted by the liver and acts on the vascular system through para-hydroxybenzoic acid mechanism downstream of tyrosine kinase receptor IGF1R (Stanley et al. 2021)[15]. Multiple signaling effectors work downstream of IGF1R, including PI3K, PKB (also known as Akt), and extracellular signal-regulated kinase (ERK, also known as mitochondrial activated protein kinase [MAPK]) (Y. Sun et al. 2016a, b). Previous studies have suggested that IGF-1 can play a role in the development and diseases of the vascular and respiratory system. Under in vitro conditions, IGF-1 stimulation can promote smooth muscle cell (SMC) growth and inhibit cell apoptosis (Sun et al. 2016a, b). This was further confirmed by another study, which suggested that IGF-1 inhibits cell apoptosis in PASMCs by promoting the expression of p38, MAPK, and inducible nitric oxide synthase (Liu et al. 2020). The chronic expression of IGF-1 in the blood vessel wall can promote the contraction of arterial blood vessels. Experiments have shown that direct stimulation of aortic smooth muscle cells with IGF-1 in vitro can increase SMC differentiation markers such as: α-SMA, calcium Regulin and SM22α. (He et al. 2021). Stimulation of SMCs with IGF-1 can increase the expression of SMC differentiation markers, such as α-SMA and elastin, and can raise the calcium levels inside these cells (Shuang et al. 2018). Studies have also found that IGF-1 plays an important role in chronic lung diseases of premature infants and newborns, such as respiratory distress syndrome and bronchopulmonary dysplasia (Dong et al. 2012; Kheirollahi et al. 2022). However, the role of IGF-1 in PAH pathogenesis, including the mechanism underlying PASMC growth, and vascular remodeling during PAH has not been completely described earlier.

Researchers have found that some non-coding RNAs are involved in the pathogenesis of PAH (Zahid et al. 2020). circRNAs are a type of non-coding RNAs. Unlike linear non-coding RNAs, circRNAs are closed single-stranded RNA molecules formed via specific splicing mechanisms; hence, they are very stable, have longer half-life, and are resistant to exonucleases (A. Huang et al. 2020). The involvement of circRNAs during PAH progression still remains unclear. In our study, we have demonstrated an interplay between circDiaph3 and IGF1R during PAH pathogenesis. Our experiments showed that inhibition of circDiaph3 in PASMC reduced IGF-1 signaling, which negatively affected PASMC proliferation and expression of SMC marker genes, such as α-SMA and Vcam1. However, the overexpression of IGF1R in PASMCs rescued the PASMC proliferation and SMC marker gene expression, which confirmed the relationship between IGF-1 signaling and circDiaph3 during PAH progression.

The PI3K/AKT/mTOR signaling pathway is an important intracellular signal transduction pathway with a wide range of cell regulatory functions and plays an important role in cell proliferation and differentiation, glucose metabolism, insulin secretion and its signal transduction (Basile et al. 2022; J. Huang et al. 2022). PI3K can transmit signals to increase cyclin synthesis, thereby promoting the progression of G1 phase, shortening the cell cycle, and causing cell proliferation and activation. PI3K is involved in the biological effects of a variety of growth factors (Wang et al. 2022). PI3k-activated Akt can activate or inhibit its downstream target proteins, NF-κB, Forkhead, m TOR, etc. through phosphorylation to promote cell survival through a variety of pathways (Cai et al. 2019; Shao et al. 2022). PI3K/AKT/mTOR signaling pathway plays a crucial role in cell growth and differentiation (Chen et al. 2022; Tewari et al. 2022). The activation of PI3K/AKT/mTOR signaling pathway is closely related to the proliferation of vascular wall cells. Inhibition of Akt activation in cells can inhibit the growth of vascular smooth muscle cells induced by growth factors. Therefore, the activation of PI3K/AKT/mTOR signaling pathway is related to VSMCs phenotypic transformation and cell proliferation.

Pulmonary hypertension is a complex and life-threatening lung disease characterized by increased pulmonary vascular resistance, leading to increased pulmonary arterial pressure. Despite the available treatment methods, the prognosis of PAH is still poor because the underlying biological mechanisms are not fully understood. Therefore, it is particularly necessary to search for potential biomarkers to promote early diagnosis and individualized treatment. In the experiment, we induced pulmonary hypertension in rats through hypoxia. The advantage of this method is that it conforms to the pathological process of clinical patients, and the pathological results of pulmonary hypertension in rats are consistent with clinical patients.

## Conclusion

Pulmonary hypertension is still incurable in clinical practice. In study, we investigated the role of circDiaph3 in the proliferation and migration of pulmonary artery smooth muscle cells during pulmonary hypertension. CircDiaph3 expression was analyzed in blood samples from patients with pulmonary hypertension and verified in PH rat model. We found that circDiaph3 increased smooth muscle cell proliferation and reduced apoptosis by regulating the PI3K/AKT/mTOR pathway through IGF1R. In conclusion, circDiaph3 plays an important role in promoting the proliferation of pulmonary artery smooth muscle cells in rats with pulmonary hypertension through IGF1R and PI3K/AKT/mTOR signaling pathways, suggesting the potential value of circDiaph3 in the treatment of pulmonary hypertension.

Our results provide a comprehensive understanding of the molecular mechanisms underlying PAH progression. However, our study still has some limitations. Although we have demonstrated that CircDiaph3 acts on IGF1R, its direct and cross-acting mechanisms still need to be further explored. In clinical studies, our baseline data collection of patients is still not perfect, and we will add more abundant and comprehensive baseline data of patients in future studies. We strongly believe that circDiaph3 can be used as molecular marker for PAH diagnosis and therapeutic target for developing therapeutic interventions in future.

### Supplementary Information

Below is the link to the electronic supplementary material.

Supplementary file1 (DOCX 2829 KB)

Supplementary file2 (XLS 53 KB)

## Data Availability

All data generated or analyzed during this study are included in this published article.

## Abbreviations

PAH: Pulmonary arterial hypertension
PVR: Pulmonary vascular resistance
PASMCs: Pulmonary artery smooth muscle cells
IGF1R: Insulin-like growth factor 1 receptor
(IL)-1: Interleukin-1
(IL)-8: Interleukin-8
(IL)-6: Interleukin-6
CHD: Coronary heart disease
SD: Sprague Dawley
PBS: Phosphate-buffered saline
α-SMA: α-Smooth muscle actin
qRT-PCR: Quantitative real-time polymerase chain reaction
PVDF: Polyvinylidene fluoride
HRP: Horseradish peroxidase
CCK8: Cell counting Kit 8
HE: Hematoxylin–eosin staining
MAPK: Mitochondrial activated protein kinase
SMC: Smooth muscle cell
PI3K: Phosphatidylinositol 3-kinase
mTOR: Mammalian target of rapamycin
α-SMA: Actin alpha 2
VCAM-1: Vascular cell adhesion molecule-1
