# Supplementary material for: CircDiaph3 influences PASMC apoptosis by regulating PI3K/AKT/mTOR pathway through IGF1R
Source: 3 Biotech. 2023 Sep 11;13(10):342. doi: 10.1007/s13205-023-03739-0 (PMC10495302; doi:10.1007/s13205-023-03739-0)

**sSupplement Table 1. Univariate analysis**

Abbreviations: PAH, pulmonary arterial hypertension

| PAH | Statistics | β (95% CI) | p-Value |
| --- | --- | --- | --- |
| AGE | 62.2 ± 11.6 | -0.4 (-0.8, -0.1) | 0.044 |
| Anteroposterior diameter of right ventricle | 26.5 ± 10.3 | 0.4 (-0.1, 0.8) | 0.143 |
| Left ventricular end contractile diameter | 34.4 ± 9.4 | -0.3 (-0.8, 0.3) | 0.363 |
| left ventricular end diastolic diameter | 48.6 ± 12.1 | -0.2 (-0.6, 0.2) | 0.410 |
| Inner diameter of main pulmonary artery | 27.6 ± 5.8 | 0.7 (-0.2, 1.5) | 0.138 |
| Inner diameter of ascending aorta | 25.0 ± 1.6 | -1.6 s(-4.6, 1.4) | 0.306 |
| Sex |  |  |  |
| Female, n (%) | 11 (73.3%) |  | 0 |
| Male, n (%) | 4 (26.7%) | 1.0 (-10.3, 12.2) | 0.870 |
| CircDiaph3 | 2.8 ± 0.8 | 6.4 (1.0, 11.9) | 0.039 |

**Supplement Figure 1. Association betweenPAH and CircDiaph3**

**
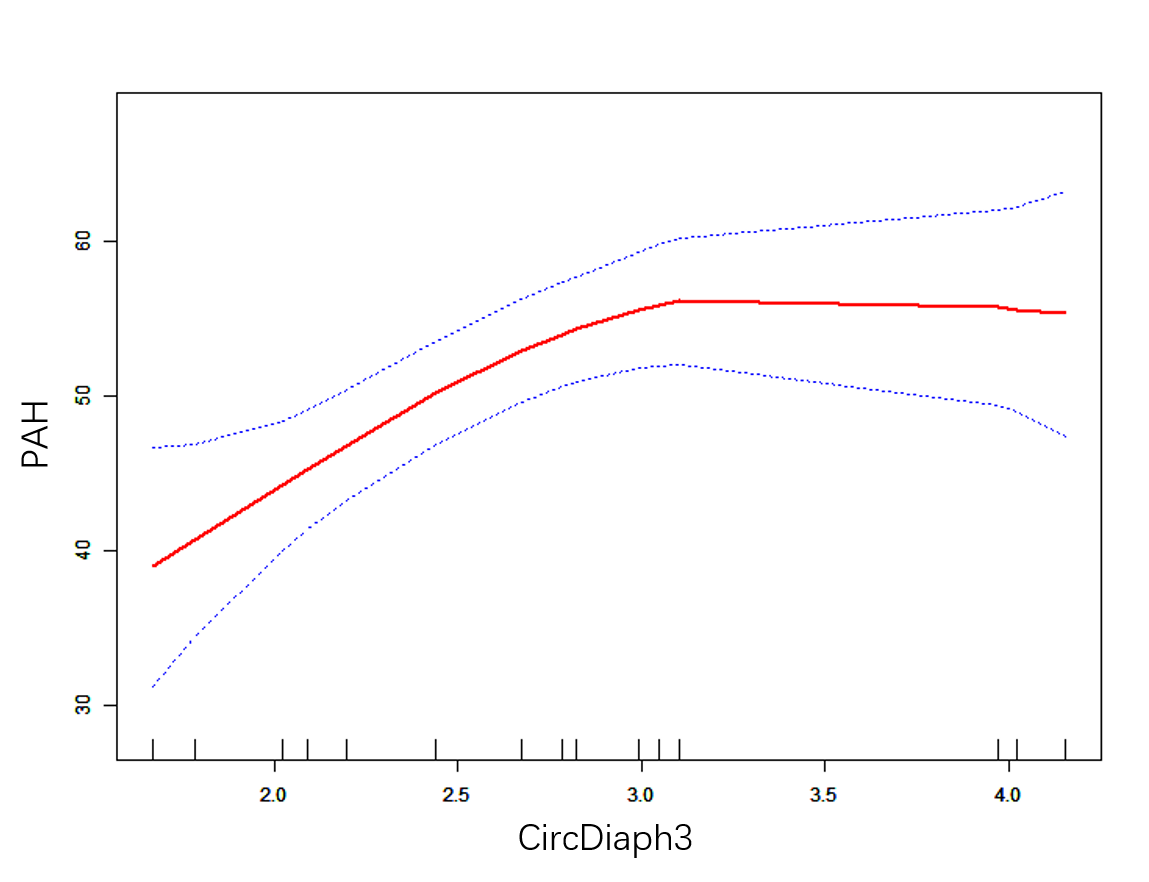
**

Abbreviations: PAH, pulmonary arterial hypertension

**Supplement Figure 2**


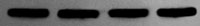
 1 2 3 4

42kDa β-actin


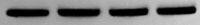


55kDa AKT


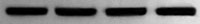
289kDa mTOR


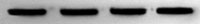
85kDa PI3K


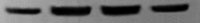
60kDa p-AKT


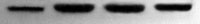
289 kDa p-mTOR


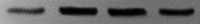
80kDa p-PI3K


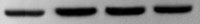
95KDa IGF1R


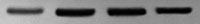
81 KDa VCAM1


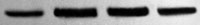
45kDa α-SMA

1：sham 2：control 3：sh-NC 4：sh-circDiaph3

**WB fig2B**

β-actin


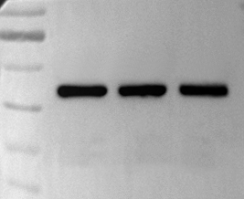

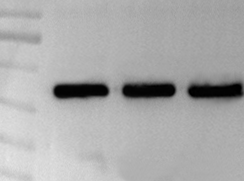

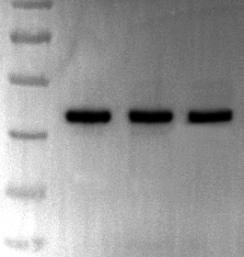


AKT


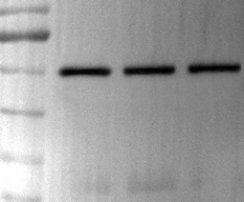

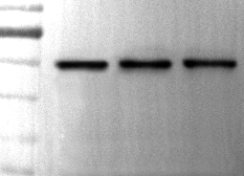

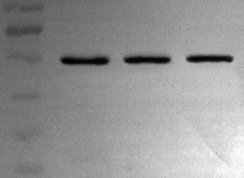


mTOR


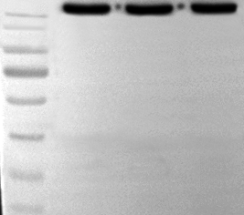

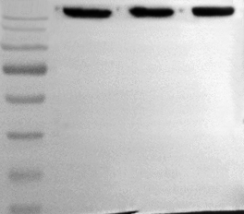

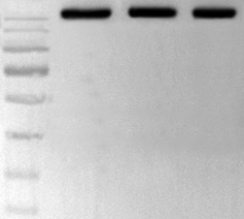


PI3K


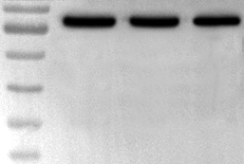

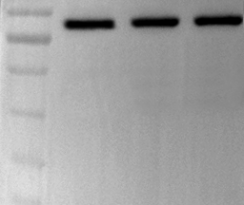

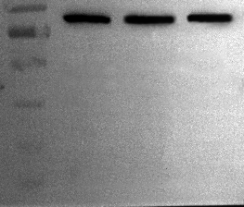


p-AKT


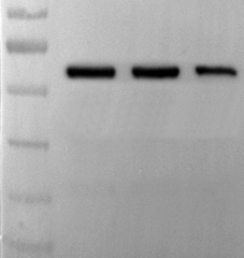

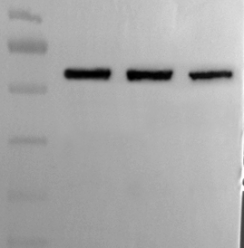

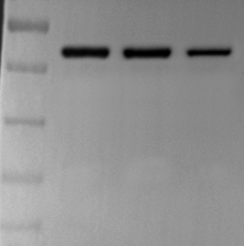


p-mTOR


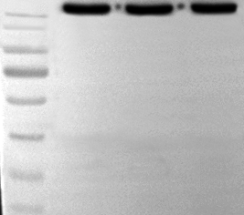

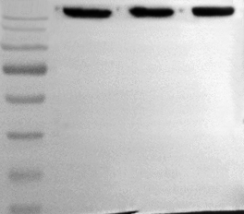

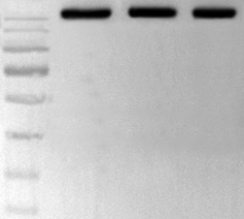


p-PI3K


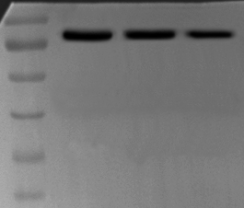

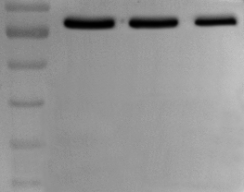

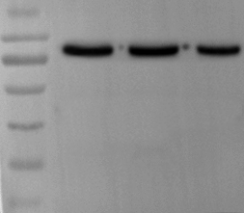


IGF1R


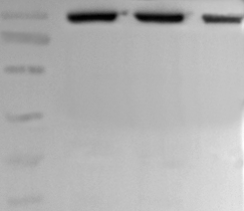

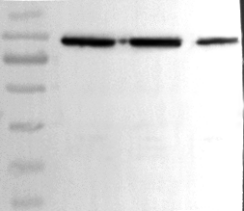

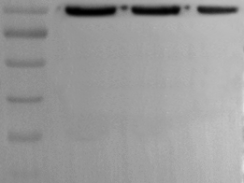


VCAM1


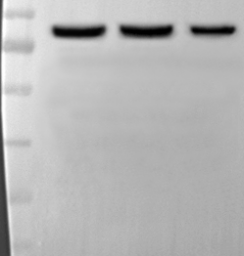

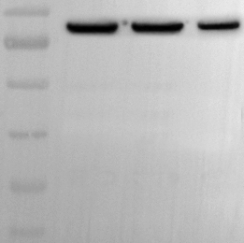

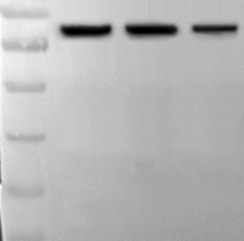


α-SMA


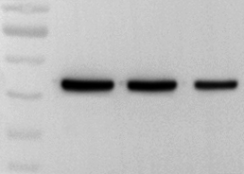

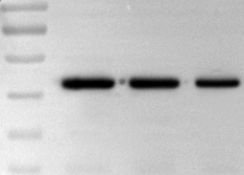

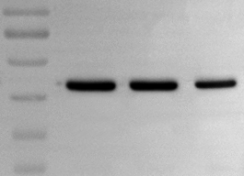


**WB fig4B**

β-actin


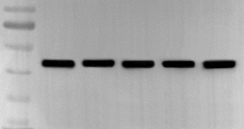

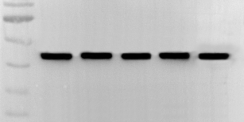

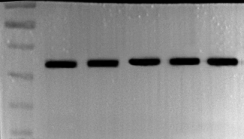


AKT


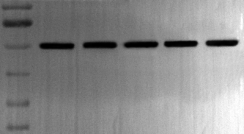

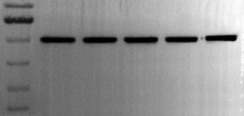

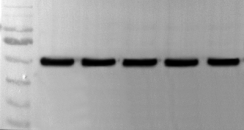


mTOR


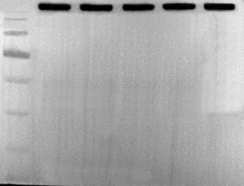

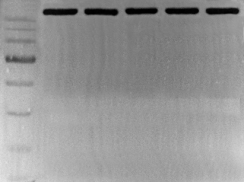

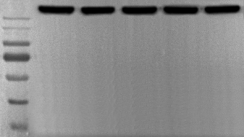


PI3K


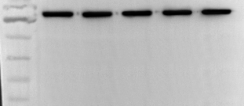

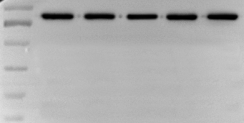

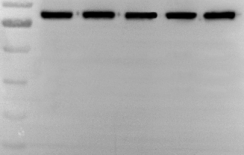


p-AKT


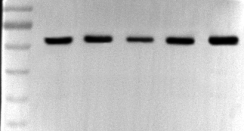

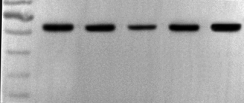

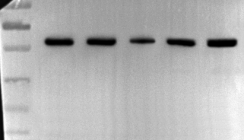


p-mTOR


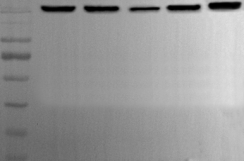

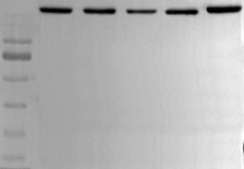

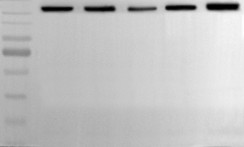


p-PI3K


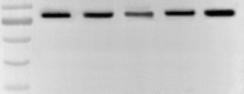

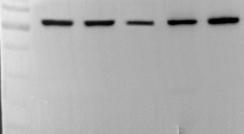

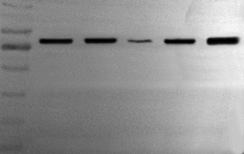


IGF1R


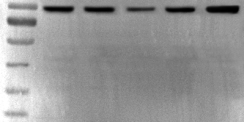

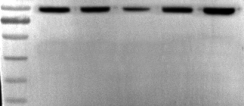

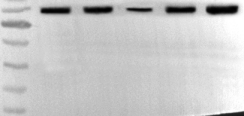


VCAM1


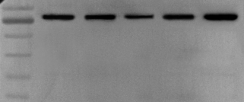

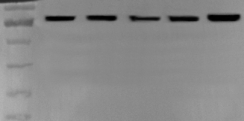

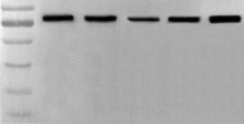


α-SMA


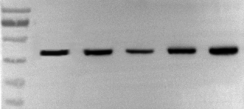

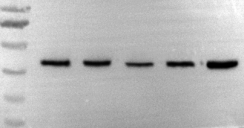

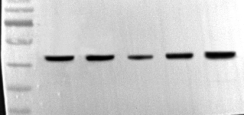


**Supplement Figure 2**

β-actin


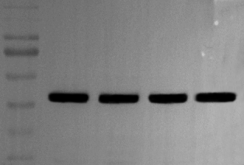

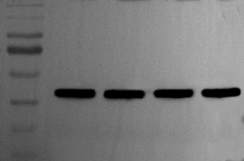

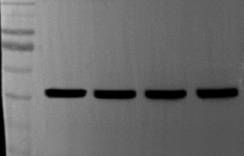


AKT


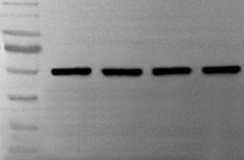

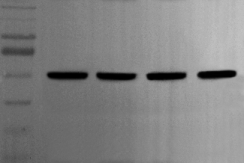

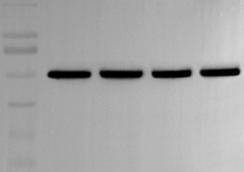


mTOR


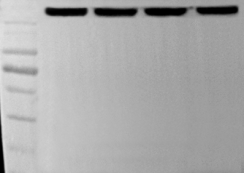

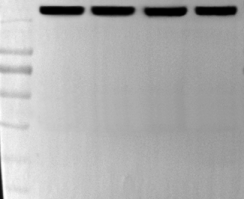

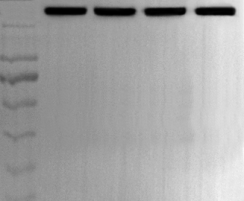


PI3K


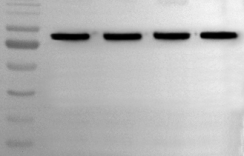

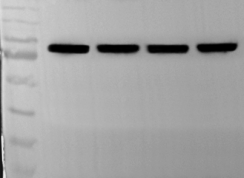

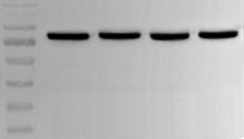


p-AKT


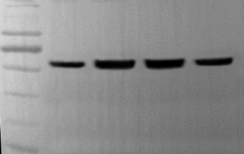

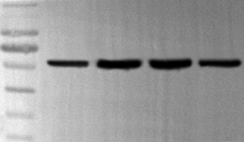

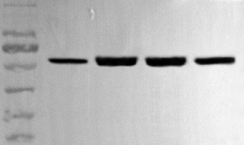


p-mTOR


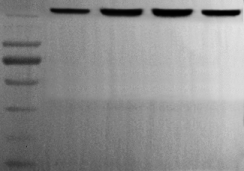

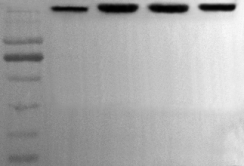

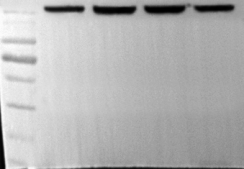


p-PI3K


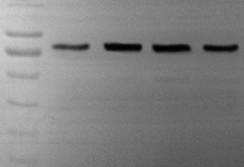

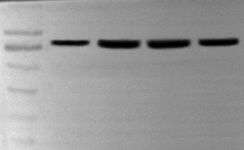

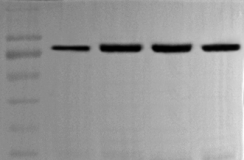


IGF1R


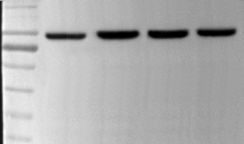

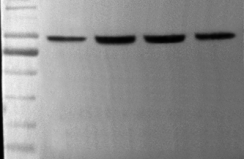

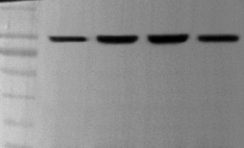


VCAM1


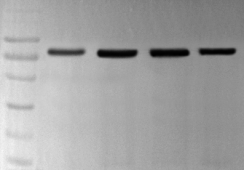

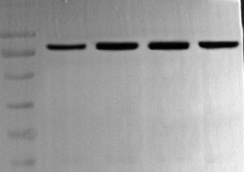

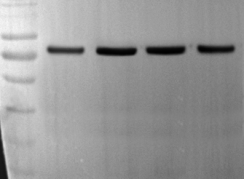


α-SMA


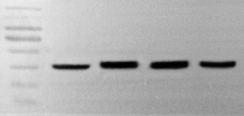

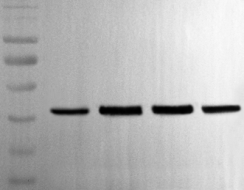

Supplement: Supplementary file 1 — Supplementary file1 (DOCX 2829 KB) [file 13205_2023_3739_MOESM1_ESM.docx]
